# Intracranial hemorrhage detection in 3D computed tomography images using a bi-directional long short-term memory network-based modified genetic algorithm

**DOI:** 10.3389/fnins.2023.1200630

**Published:** 2023-07-04

**Authors:** Jewel Sengupta, Robertas Alzbutas, Przemysław Falkowski-Gilski, Bożena Falkowska-Gilska

**Affiliations:** ^1^Kaunas University of Technology, Kaunas, Lithuania; ^2^Faculty of Electronics, Telecommunications and Informatics, Gdansk University of Technology, Gdansk, Poland; ^3^Specialist Diabetes Outpatient Clinic, Olsztyn, Poland

**Keywords:** bi-directional long short-term memory network, computed tomography, genetic algorithm, gradient local ternary pattern, intracranial hemorrhage detection, Tamura features, region of interest

## Abstract

**Introduction:**

Intracranial hemorrhage detection in 3D Computed Tomography (CT) brain images has gained more attention in the research community. The major issue to deal with the 3D CT brain images is scarce and hard to obtain the labelled data with better recognition results.

**Methods:**

To overcome the aforementioned problem, a new model has been implemented in this research manuscript. After acquiring the images from the Radiological Society of North America (RSNA) 2019 database, the region of interest (RoI) was segmented by employing Otsu’s thresholding method. Then, feature extraction was performed utilizing Tamura features: directionality, contrast, coarseness, and Gradient Local Ternary Pattern (GLTP) descriptors to extract vectors from the segmented RoI regions. The extracted vectors were dimensionally reduced by proposing a modified genetic algorithm, where the infinite feature selection technique was incorporated with the conventional genetic algorithm to further reduce the redundancy within the regularized vectors. The selected optimal vectors were finally fed to the Bi-directional Long Short Term Memory (Bi-LSTM) network to classify intracranial hemorrhage sub-types, such as subdural, intraparenchymal, subarachnoid, epidural, and intraventricular.

**Results:**

The experimental investigation demonstrated that the Bi-LSTM based modified genetic algorithm obtained 99.40% sensitivity, 99.80% accuracy, and 99.48% specificity, which are higher compared to the existing machine learning models: Naïve Bayes, Random Forest, Support Vector Machine (SVM), Recurrent Neural Network (RNN), and Long Short-Term Memory (LSTM) network.

## 1. Introduction

Intracranial hemorrhage is a critical disease that causes severe disability and even death (Morotti et al., 2018; Remedios et al., 2020). Intracranial hemorrhage is caused by various pathologies, such as cerebral aneurysms, dural arteriovenous fistulas, hypertension, vasculitis, trauma, cerebral amyloid angiopathy, cerebral arteriovenous malformation, venous sinus thrombosis, and hemorrhagic conversion of ischemic infarction (Cheruiyot et al., 2021). On the other hand, hemorrhagic disease is caused by the elimination of path interaction and excessive leakage of blood in the vessels. The main risk factors for hemorrhagic disease are leakage in veins, infected blood vessel walls, high blood pressure, and head trauma. CT is an effective and non-invasive imaging technique for recognizing intracranial hemorrhage when compared to other imaging techniques such as histology, x-rays, MRIs, ultrasound, etc. (Duperron et al., 2019). In addition, hemorrhage is easily detected on CT images because human blood has a high density compared to brain tissue, but the density is lower than bone (Karki et al., 2020). The hemorrhage clots on the CT images are based on external factors like volume, position, slice intensity, scanning angle, and density.

Accurate detection of bleeding is crucial for physicians to perform clinical interventions (Lee et al., 2019; Patel et al., 2019). However, the manual intervention carried out by physicians is a time-consuming task. Therefore, an automated intracranial hemorrhage model is essential (Huang et al., 2019; Sage and Badura, 2020). In the last decades, several artificial intelligence and deep learning algorithms have been successfully employed for medical image analysis, such as breast cancer detection, skin cancer detection, grading of diabetic retinopathy, etc. Also, artificial intelligence (AI) algorithms ensure proper detection to facilitate timely diagnosis, which significantly reduces the mortality rate. There are already several algorithms for intracranial hemorrhage detection based on deep learning models. However, most of the existing models face difficulties in segmenting intracranial hemorrhage regions in 3D brain scans because of their scarce nature (Raghavendra et al., 2021). Additionally, both the validation and training datasets are limited in the existing reported studies. In most of the previous studies, the developed model’s performance was only validated at the scan level rather than at the slice-by-slice verification level. In this manuscript, an efficient and accurate model for intracranial hemorrhage recognition was implemented.

The main contributions are as follows:

- First, we used Otsu’s thresholding technique for region segmentation in the collected brain images and performed hybrid feature extraction (GLTP descriptor and Tamura features) to extract discriminative vectors. The hybrid feature extraction significantly reduced the semantic gap between the feature subsets, which helped to obtain significant classification performance.
- Next, we proposed a modified genetic algorithm for feature optimization, where an infinite scheme is used to reduce redundancy in the genetic algorithm. The feature optimization effectively decreases the computational complexity and time of the proposed framework.
- Then, we used a Bi-LSTM network in order to classify intracranial hemorrhage types: subdural, intraparenchymal, subarachnoid, epidural, and intraventricular. The efficacy of the Bi-LSTM-based modified genetic algorithm was tested using evaluation metrics such as the Dice coefficient, Jaccard coefficient, Matthews Correlation Coefficient (MCC), accuracy, the F1 Score, specificity, and sensitivity.

The organization of this study is as follows: studies related to intracranial hemorrhage are reviewed in Section 2. Next, the mathematical explanations and the simulation results of the Bi-LSTM-based modified genetic algorithm are given in Sections 3 and 4, respectively. The conclusion of this study is given in Section 5.

## 2. Literature review

Kumar et al. (2022) introduced an entropy-based segmentation framework for effective intracranial hemorrhage detection utilizing CT images. The developed framework includes a skull removal model, an edge-based active contour model, a thresholding model, and a fuzzy C-Means (FCM) algorithm for automatic cluster selection. While the incorporation of several models increases the computational complexity of the framework. Vrbančič et al. (2019) used transfer learning with the grey wolf optimization algorithm to detect hemorrhage in the CT brain images. The numerical outcomes show that the presented method outperforms the conventional methods by using different evaluation measures, but it has a computational problem in finding the best possible solutions. Wang et al. (2020) used the U-Net model for intracranial hemorrhage detection utilizing CT images. Alis et al. (2022) implemented an RNN model to detect intracranial hemorrhage on non-contrast head CT images. On the other hand, U-Net and RNN models were suitable for hemorrhage detection but computationally expensive. Kuo et al. (2019) used Deep Convolutional Neural Network (D-CNN) for intracranial hemorrhage detection on CT images. As specified above, the CNN model needs an enormous amount of data for model training, which is computationally expensive.

Li et al. (2021) developed U-Net for the automatic detection and segmentation of intracranial hemorrhage strokes in 3D-CT brain images. Additionally, adversarial training was adopted to enhance segmentation accuracy. The experimental evaluations demonstrated the robustness, effectiveness, and advantages of the developed U-Net model in intracranial hemorrhage lesion diagnosis. However, the implemented U-Net model requires larger amounts of data to attain significant classification results. Burduja et al. (2020) utilized the Bi-LSTM network and ResNeXt-10 model for feature selection and intracranial hemorrhage subtype classification. In this literature, human evaluations were conducted to compare the accuracy level of the developed model with that of highly trained doctors. Deep learning models like ResNeXt-10 were computationally costly because they required higher-end graphics processing units to process the larger unstructured databases. Mansour and Aljehane (2021) integrated Kapur’s threshold with the elephant herd optimizer for region segmentation. Next, the Inception V4 network was implemented for vector extraction, and then classification was performed employing a multi-layer perceptron. The extensive experiment showed the effectiveness of the presented model, and furthermore, the results were evaluated under different dimensions.

Wang et al. (2021) used 2D CNN for precise lesion detection and subtype classification of intracranial hemorrhage. The experimental results confirmed that the developed 2D CNN model achieved robust and high classification performance, but it was computationally costly. Imran et al. (2021) introduced a fully convolutional network named U-Net for effective intracranial hemorrhage lesion segmentation and classification. As mentioned earlier, deep learning models like U-Net are computationally costly. In addition, Anupama et al. (2022) first utilized the Gabor filtering technique for removing noise from the raw brain images. Further, a grab-cut with a synergistic deep-learning model was used for intracranial hemorrhage segmentation. Finally, a CNN model was applied to classify the subtypes of hemorrhage. In the numerical analysis section, the developed model has achieved higher classification results in terms of specificity, recall, and accuracy. Lee et al. (2020) presented an artificial neural network for effective lesion detection in intracranial hemorrhage. Simulation outcomes demonstrated that the developed model effectively reduced the diagnosis time with good diagnostic performance, but that it has high variance and bias when processing unbalanced databases.

Gautam and Raman (2021) have performed pre-processing operations such as normalization and contrast enhancement to improve the quality of the collected raw CT images. Then, the denoised brain images were fed to the 13-layer CNN model to classify the types of strokes. As mentioned earlier, the CNN model requires larger amounts of data to obtain superior results. Hssayeni et al. (2020) utilized a U-Net model to segment intracranial hemorrhage lesions from 3D CT images. As shown in the resulting segment, the U-Net model has higher segmentation performance using Jaccard and Dice coefficients with 5-fold cross-validation. Ye et al. (2019) integrated 3D CNN and RNN for better detection of intracranial hemorrhage diseases. Hence, the presented model effectively classified five subtypes, such as subarachnoid, epidural, intraventricular, subdural, and cerebral parenchymal, in 3D CT images. However, the hybrid deep learning models were computationally complex and consumed more computational time to process the data (Wang et al., 2022, 2023). To overcome the above issues and improve intracranial hemorrhage detection, a new Bi-LSTM-based modified genetic algorithm has been introduced in this research study.

## 3. Methods

**Figure 1:**
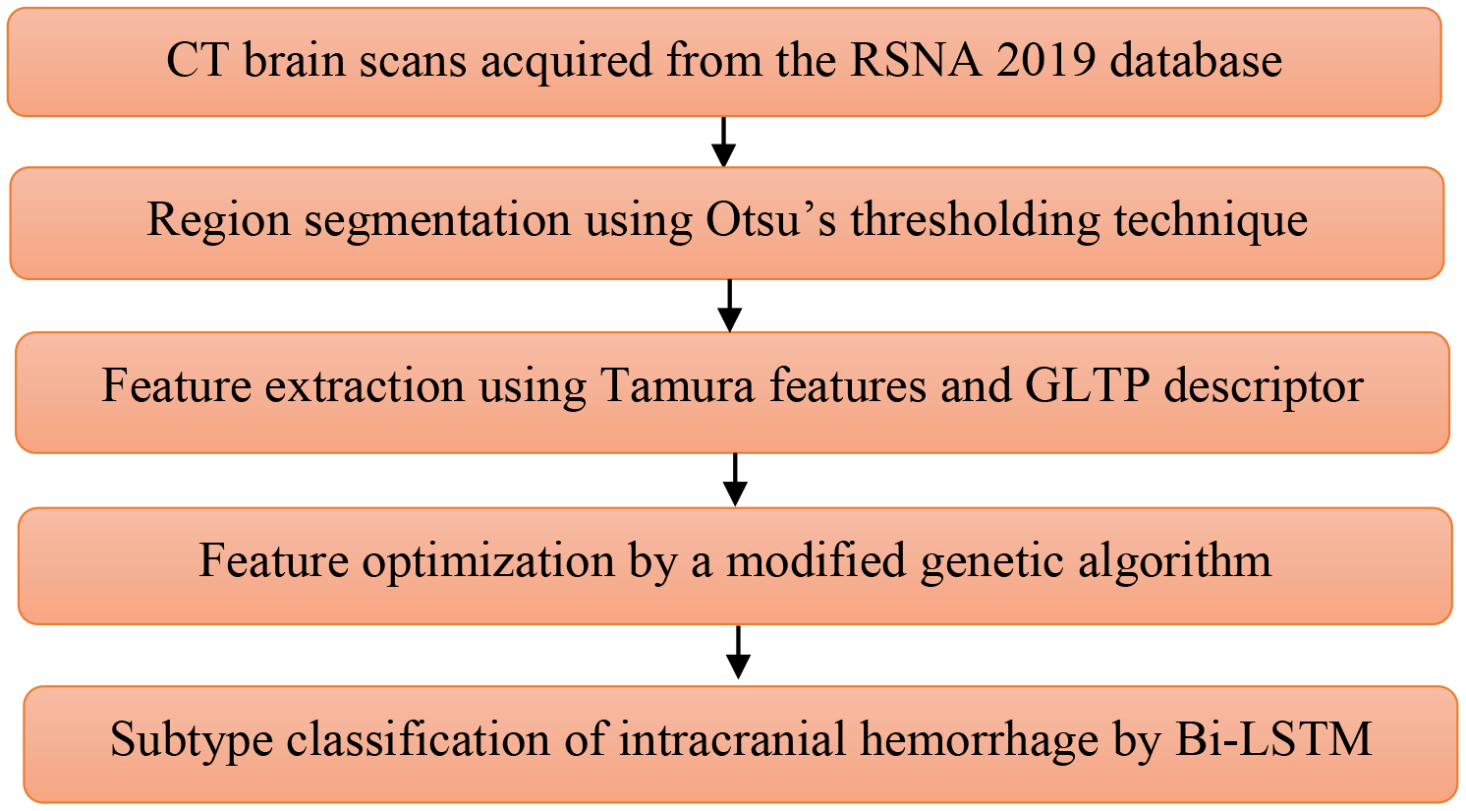
Flow diagram of the proposed framework.

In this study, the proposed framework has five steps, such as **Database Description:** RSNA 2019 database, **Region Segmentation:** Otsu’s thresholding technique, **Feature Extraction:** GLTP descriptors and Tamura features, **Feature Optimization:** modified genetic algorithm, and **Classification:** Bi-LSTM network. The flow diagram of the proposed framework is shown in Figure 1.

### 3.1. Database description

**Figure 2:**
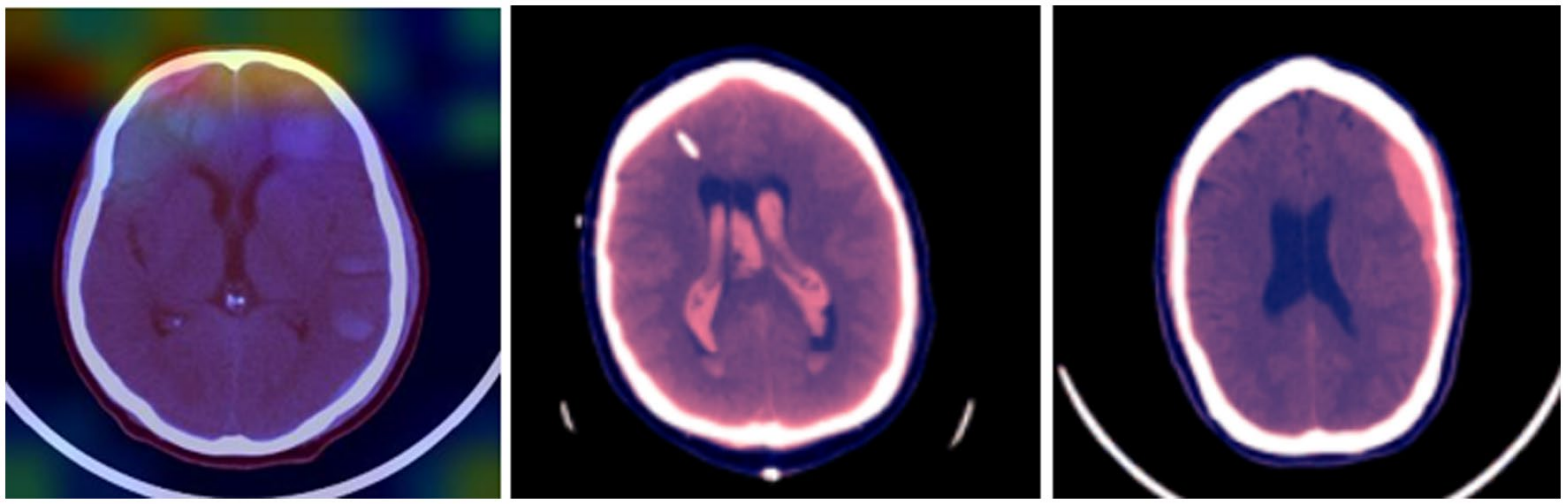
Recorded 3D brain images.

The effectiveness of the developed Bi-LSTM-based modified genetic was tested on an online benchmark database. The undertaken database has 25,272 images with 870,301 brain slices, and the acquired images were then labeled by the annotators as five classes: subdural, intraparenchymal, subarachnoid, epidural, and intraventricular. In the acquired database, the annotators did not have details about the patient’s medical history, the acuity of their symptoms, prior examinations, OR the patient’s age. The brain images of the acquired database are shown in Figure 2.

### 3.2. Region segmentation

After the acquisition of the brain images, region segmentation was performed by employing Otsu’s thresholding, which helped determine the maximum separability of the classes such as subdural, intraparenchymal, subarachnoid, epidural, and intraventricular (Feng et al., 2017). In this technique, the selected pixel intensity value of the image is related to the average pixel intensity value to improve the segmentation results. First, the acquired 3D brain scans were portioned into two binary regions, i.e., dark $\;T_{1}$ and light $\;T_{0}$ regions, which were mathematically represented in Eqs 1, 2, with l being represented as histogram bins.

(1) $$T_{0}=\left\{0,\;\;1,\;\;\ldots .\;\;,\;\;t\right\}$$

(2) $$T_{1}=\left\{t,\;\;t+1,\;\;\ldots .\;\;,\;\;l-1,\;\;l\right\}$$

In this scenario, the threshold value was set as t=0.5 , where it effectively discriminated between the overlapping intracranial hemorrhage classes (Al-Rahlawee and Rahebi, 2021; Tan et al., 2021; Dutta et al., 2022). The optimal threshold value t=0.5 was found by minimizing the weight group variance based on the distinct group’s p(i) probability, and it was mathematically represented in Eq. 3.

(3) $$p\left(i\right)=\frac{number\;\left\{\left(r,c\right)|image\left(r,c\right)=i\right\}}{\left(r,c\right)}$$

In the 3D brain images, the variance $\;\sigma_{b}^{2}\left(t\right)\;and\;\sigma_{f}^{2}\left(t\right)$ , mean $\;\mu_{b}\left(t\right)\;and\;\mu_{f}\left(t\right)$ , and weight $\;w_{b}\left(t\right)\;and\;w_{f}\left(t\right)$ of the light and dark regions $T_{0}$ and $T_{1}$ were computed using Eqs 4, 5.

(4) $$\begin{array}{l} w_{b}\left(t\right)=\sum_{i=1}^{t}\;p\left(i\right),\mu_{b}\left(t\right)=\frac{\sum_{i=1}^{t}i\times p\left(i\right)}{w_{b}\left(t\right)}\;and\;\\ \sigma_{b}^{2}\left(t\right)=\frac{\sum_{i=1}^{t}{\left(i-\mu_{b}\left(t\right)\right)}^{2}\times p\left(i\right)}{w_{b}\left(t\right)} \end{array}$$

(5) $$\begin{array}{c} w_{f}\left(t\right)=\sum_{i=t+1}^{l}p\left(i\right),\mu_{f}\left(t\right)=\frac{\sum_{i=t+1}^{l}i\times p\left(i\right)}{w_{f}\left(t\right)}\;and\;\\ \sigma_{f}^{2}\left(t\right)=\frac{\sum_{i=t+1}^{l}{\left(i-\mu_{f}\left(t\right)\right)}^{2}\times p\left(i\right)}{w_{f}\left(t\right)} \end{array}$$

The optimal threshold was found with lower class variance $\;\sigma_{w}^{2}$ , and was mathematically represented in Eq. 6. After the segmentation of the intracranial hemorrhage regions, feature extraction was performed using GLTP descriptors and Tamura features.

(6) $$\sigma_{w}^{2}=w_{b}\left(t\right)\times \sigma_{b}^{2}\left(t\right)+w_{f}\left(t\right)\times \sigma_{f}^{2}\left(t\right)$$

### 3.3. Extraction of discriminative vectors

After region segmentation, the discriminative vectors were extracted by implementing the GLTP descriptor and Tamura features. In this manuscript, three Tamura features $f_{Tamura}$ like directionality, contrast, and coarseness were implemented for the extraction of discriminative vectors from the segmented regions. First, directionality generated the edge probability of the image histograms by quantizing the edge angles, with this procedure helping to sharpen the image edges. Second, contrast-enhanced the gray level in the segmented regions by distributing the pixel intensity value. Third, coarseness mainly relied on the texture scale and repetition rates in the brain images to find patterns with different structures. A total of 3,492 vectors were extracted from the segmented region utilizing the Tamura features (Karmakar et al., 2017; Tao and Lu, 2018; Das et al., 2019; Yu et al., 2020).

In addition to this, GLTP is a texture descriptor that encodes the local texture of the segmented images by quantizing the pixel intensity value into three discrimination levels and by estimating the gradient magnitude. The GLTP ensures the texture patterns even under conditions of illumination variations and random noise. First, the horizontal and vertical approximations ( $G_{i},G_{j}$ ) of the segmented images were obtained using the Sobel Feldman operator. Then, the gradient magnitude $G_{i,j}$ of each brain image was obtained by integrating $G_{i}$ and $G_{j}$ – this was mathematically represented in Eq. 7.

(7) $$G_{i,j}=\sqrt{G_{i}^{2}+G_{j}^{2}}$$

A threshold value t was used around the Center Gradient $\;G_{c}$ value of 3×3 neighborhood image pixels to distinguish both smooth and highly textured regions from $\;G_{i,j}$ , − this was mathematically specified in Eq. 8 (Holder and Tapamo, 2017; Fekri-Ershad, 2020).

(8) $$S_{GLTP}\left(G_{c},G_{i,j}\right)=\left\{\begin{array}{cc} -1 & \;G_{i,j}<G_{c}-t\\ 0 & \;G_{c}-t\leq G_{i,j}\leq G_{c}+t\\ +1 & \;G_{i,j}>G_{c}+t \end{array}\right\}$$

Where $S_{GLTP}$ is the quantized value of the neighborhood image pixels. The gradient values below $G_{c}-t$ were quantized to −1, gradient values above $G_{c}+t$ were quantized to 1 and then the gradient values falling between $G_{c}+t$ and $\;G_{c}-t$ were quantized to zero. The obtained three-level discrimination coding was high-dimensional, and further, $S_{GLTP}$ was categorized into –ve $N_{GLTP}$ and + ve $P_{GLTP}$ decimal codes, which were mathematically given in Eqs 9, 10.

(9) $$N_{GLTP}=\overset{7}{\underset{i=0}{\sum }}S_{N}\left(S_{GLTP}\left(i,j\right)\right)\times 2^{i,j},S_{N}\left(v\right)=\left\{\begin{array}{cc} 1 & if\;v<0\\ 0 & else \end{array}\right\}$$

(10) $$P_{GLTP}=\overset{7}{\underset{i=0}{\sum }}S_{P}\left(S_{GLTP}\left(i,j\right)\right)\times 2^{i,j},S_{P}\left(v\right)=\left\{\begin{array}{cc} 1 & if\;v>0\\ 0 & else \end{array}\right\}$$

In the next step, the GLTP histogram values were computed from $N_{GLTP}$ and $\;P_{GLTP}$ for each brain image m×n , as mentioned in Eqs 11, 12.

(11) $$H_{N_{GLTP}}\left(\tau \right)=\overset{\frac{M}{m}}{\underset{r=1}{\sum }}\overset{\frac{N}{n}}{\underset{c=1}{\sum }}f\left(N_{GLTP}\left(r,c\right),\tau \right)$$

(12) $$H_{P_{GLTP}}\left(\tau \right)=\overset{\frac{M}{m}}{\underset{r=1}{\sum }}\overset{\frac{N}{n}}{\underset{c=1}{\sum }}f\left(P_{GLTP}\left(r,c\right),\tau \right)$$

$$\mathrm{where}\;f\left(\alpha ,\tau \right)=\{\begin{array}{cc} 1 & if\;\alpha =\tau \\ 0 & else \end{array}$$

Where (r,c) denote rows and columns in the GLTP-encoded images, (M,N) specify the width and height of the images, and α=τ represents the GLTP code, which ranges between zero and 255. Finally, $H_{N_{GLTP}}$ and $H_{P_{GLTP}}$ values were integrated to generate the final vectors $\;F_{GLTP}$ . A total of 1,926 vectors were extracted using the GLTP texture descriptor. By using the feature level fusion technique, the extracted vectors of the GLTP descriptor and Tamura features were integrated, and further dimensionality reduction was carried out by employing a modified genetic algorithm.

### 3.4. Vector optimization

After extracting 5,418 discriminative vectors, optimization was carried out by implementing a modified genetic algorithm. In the existing research studies, a conventional genetic algorithm was used to determine the relevant vectors for disease classification. In recent decades, many variations of genetic operations have been used to further improve optimization performance. An extensively utilized method in the traditional genetic algorithm is entropy, which measures the database homogeneities to identify the mutual information among the extracted vectors, which helps in determining the active vectors.

However, a modified genetic algorithm uses a simple entropy function to find the active vectors. In the proposed algorithm, the conditional entropy value was determined for both the output vectors and the regularized vectors based on an infinite feature selection technique. The implemented algorithm aims at identifying the maximum relevance between the output vectors and the regularized vectors, which reduces the redundancy within the regularized vectors. In a modified genetic algorithm, the initial population is equal to the subset of the regularized vectors that are assumed to be the active vectors of the pre-defined outputs. In addition, the fitness function is determined based on the entropy measure by improving the mutual state of the conditional entropy function between the output and the regularized vectors, as mentioned in Eq. 13.

(13) $$Fitness=\left(\alpha \times \gamma \right)+\beta \times \left(\frac{\left|T_{f}|-|l_{c}\right|}{\left|l_{c}\right|}\right)$$

**Figure 3:**
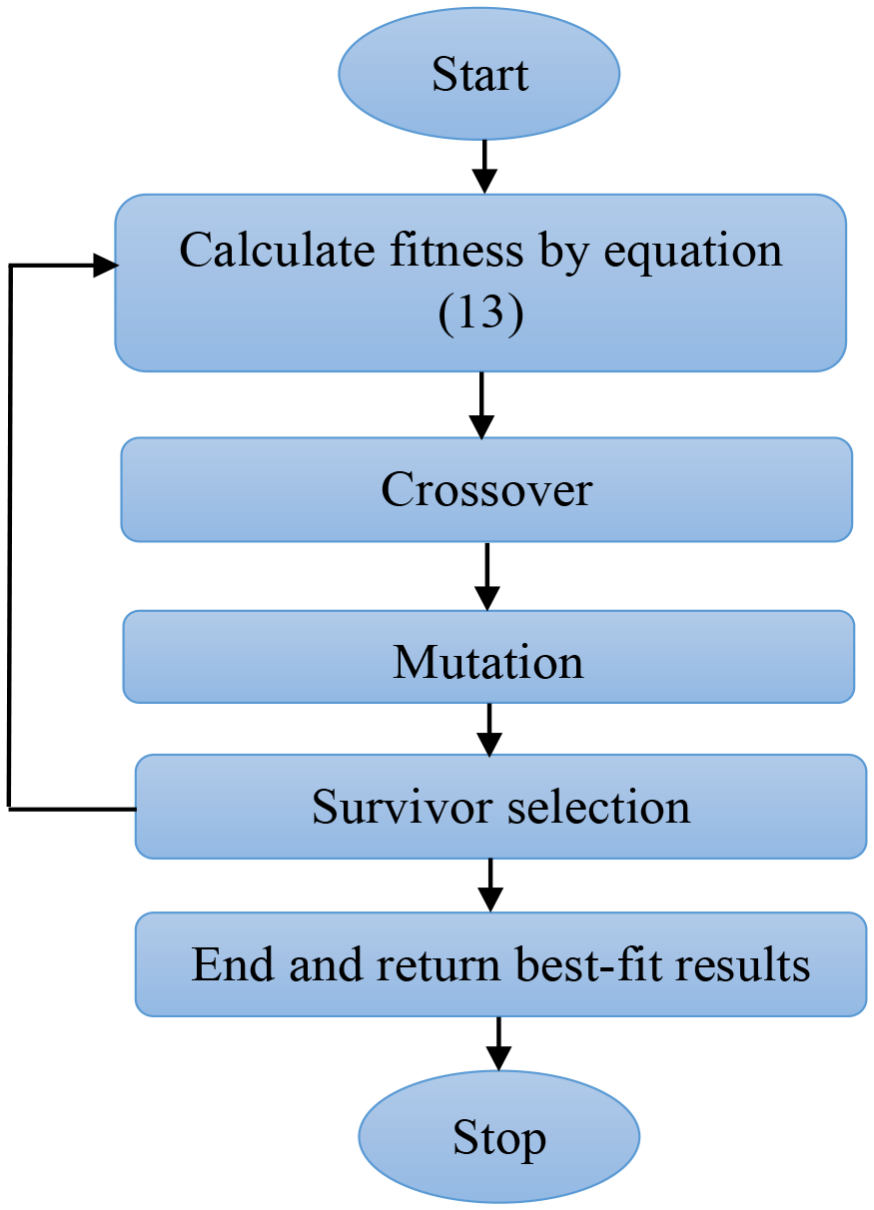
Flow diagram of the modified genetic algorithm.

The modified genetic algorithm stops when it reaches the maximum number of generations, which is 100. γ indicates the classification accuracy, αϵ[0,1] , β=1−α , $T_{f}$ indicate the extracted vectors, and $l_{c}$ represents the chromosome length. In addition, the crossover operations improved the diversity of genetics to identify the active regularized vectors (Mirjalili, 2019; Mirjalili et al., 2020; Katoch et al., 2021). The flow diagram of the modified genetic algorithm is specified in Figure 3.

**Figure 4:**
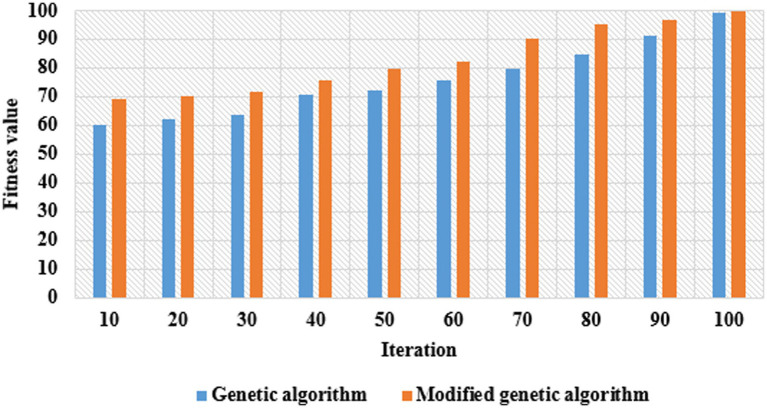
Fitness comparison between the genetic algorithm and the modified genetic algorithm.

In a modified genetic algorithm, the selection operations performed for identifying the active vectors are performed by reducing the redundancy based on the fitness function. The assumed parameters of the modified genetic algorithm are: mutation function is 0.1, population type is bit string, generation is 100, fitness function is entropy, elite count is 2, crossover function is 0.80, and population size is equal to the extracted vector length. From the extracted 5,418 vectors, a total of 2,932 vectors were selected for classification. The fitness comparison between the genetic algorithm and the modified genetic algorithm is shown in Figure 4.

### 3.5. Classification using the bi-LSTM network

**Figure 5:**
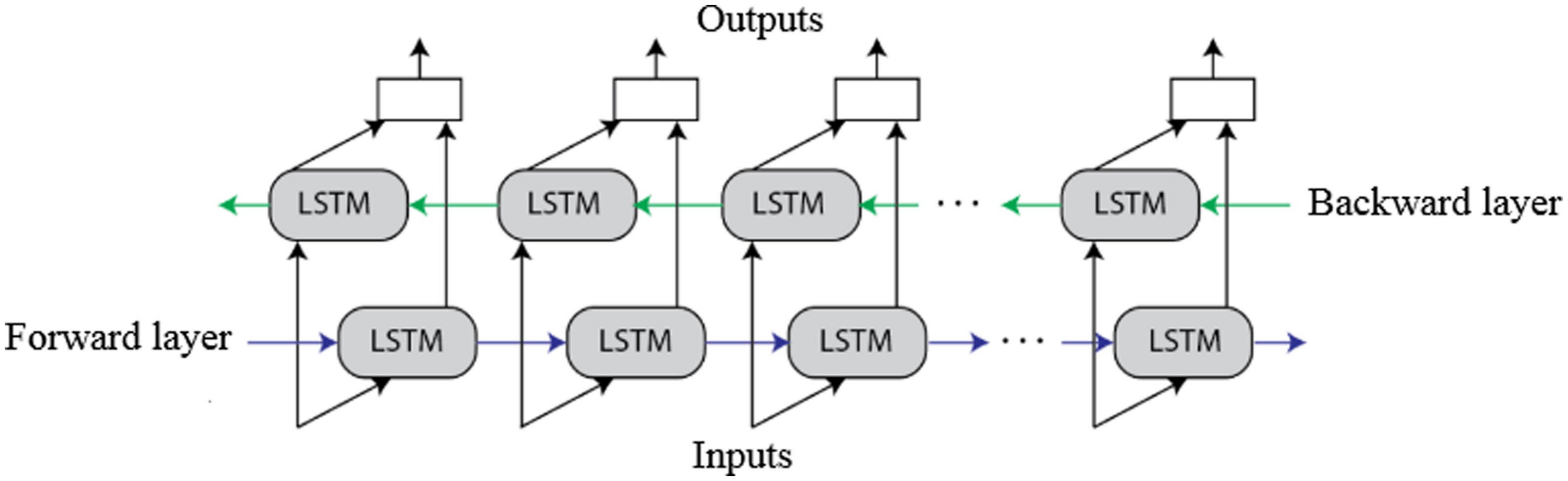
The architecture of the Bi-LSTM network.

In the final phase, the 2,932 selected vectors were fed to the Bi-LSTM network to categorize the subtypes of intracranial hemorrhage: subdural, intraparenchymal, subarachnoid, epidural, and intraventricular. The LSTM is an updated version of the RNN, and it uses memory cells to control three gates: input, output, and forget gates. This helps store the temporal state. In a conventional LSTM network, the input and output gates are utilized to handle the input and output flows of the memory cells. Further, the forget gate is connected to the memory cells to transmit the output information from the current neuron to the subsequent neurons. The information is stored in the memory cells, while the input has higher activation. Additionally, the information is transferred to the next neuron while the output has higher activation. The LSTM gates input $\;i_{t}$ , forget $\;f_{t}$ , cell $\;c_{t}$ , and output gate $\;o_{t}$ are mathematically represented in Equations (14–17). The architecture of the Bi-LSTM network is shown in Figure 5 (Alhussein et al., 2020; Shahid et al., 2020).

(14) $$i_{t}=\sigma \left(W_{ih}h_{t-1}+W_{ia}a_{t}+b_{i}\right)$$

(15) $$f_{t}=\sigma \left(W_{fh}h_{t-1}+W_{fa}a_{t}+b_{f}\right)$$

(16) $$c_{t}=f_{t}⊙c_{t-1}+i_{t}⊙\tanh\left(W_{ch}h_{t-1}+W_{ca}a_{t}+b_{c}\right)$$

(17) $$o_{t}=\sigma \left(W_{oh}h_{t-1}+W_{oa}a_{t}+b_{0}\right)$$

Where, ⊙ denotes pointwise multiplication, W and b are work coefficient values, $a_{t}=A\left[t,.\right]\in {\mathbb{R}}^{F}$ denote quasi-periodic vectors, $\;h_{t-1}$ is the output of the previous LSTM unit, tanh(.) denotes a hyperbolic tangent function, and σ(.) indicates a sigmoid function. The output of the LSTM $h_{t}$ is mathematically expressed in Eq. 18.

(18) $$h_{t}=o_{t}⊙\tanh\left(c_{t}\right)$$

The element $\;h_{t}$ contains information about the previous time steps of an output gate and a cell state. The cell state $\left\{c_{t}|t=1,2,..T\right\}$ learns memory information of $a_{t}=A\left[t,.\right]\in {\mathbb{R}}^{F}$ for a longer and shorter period based on dependency relations. In this work, the Bi-LSTM was implemented to address the concerns of the conventional LSTM, where it perfectly works on the previous content, but failed to use future content. In the Bi-LSTM network, the input flows in both forward and backward directions that helps in preserving the future and past information. The parameters considered in the Bi-LSTM network are as follows: maximum epochs are 100, execution environment is graphics processing units, gradient threshold is one, learning rate is 0.001, and batch size is 27. Hence, the extensive experimental investigation of the Bi-LSTM-based modified genetic algorithm is presented in the next section.

## 4. Simulation results

In this study, the Bi-LSTM-based modified genetic algorithm was analyzed in the Matlab 2020 software environment on a computer with 128 GB of RAM, with a Quadro K1200 CUDA device, a 4 TB hard disk, a 3.70GHz Intel ® Xenon ® central processing unit (E5-1630 v4), and a Windows 10 (64-bit) operating system. The effectiveness of the Bi-LSTM-based modified genetic algorithm was validated in terms of the Dice coefficient, Jaccard coefficient, MCC, accuracy, specificity, F1 score, and sensitivity. In this application, the Dice coefficient was used to compare the pixel-wise agreement between ground truth and a segmented region. Then, the Jaccard coefficient ranged from zero to one, where one shows perfect region overlap and zero indicates no overlap. The mathematical formulas of the Dice and Jaccard coefficients are depicted in Eqs 19, 20.

(19) $$Dicecoefficient=\frac{2TP}{2TP+FP+FN}\times 100$$

(20) $$Jaccardcoefficient=\frac{TP}{TP+FP+FN}\times 100$$

In addition to this, evaluation metrics such as MCC, accuracy, specificity, F1 Score, and sensitivity were utilized to analyze the classification performance of the Bi-LSTM-based modified genetic algorithm, where FN, TN, FP, and TP denote false negative, true negative, false positive, and true positive values. The mathematical representation of the MCC, accuracy, specificity, F1 Score, and sensitivity is specified in Eqs 21–25.

(21) $$MCC=\frac{TP\times TN-FP\times FN}{\sqrt{\left(TP+FP\right)\left(TP+FN\right)\left(TN+FP\right)\left(TN+FN\right)}}\times 100$$

(22) $$Accuracy=\frac{TP+TN}{TP+TN+FP+FN}\times 100$$

(23) $$Specificity=\frac{TN}{TN+FP}\times 100$$

(24) $$F1-score=\frac{2TP}{FP+2TP+FN}\times 100$$

(25) $$Sensitivity=\frac{TP}{TP+FN}\times 100$$

Where TP indicates that the intracranial hemorrhage regions are accurately classified as the intracranial hemorrhage regions, TN indicates that the healthy regions are accurately classified as the healthy regions, FP indicates that the intracranial hemorrhage regions are classified as the healthy regions, and finally, FN denotes that the healthy regions are classified as the intracranial hemorrhage regions.

### 4.1. Quantitative investigation

**Figure 6:**
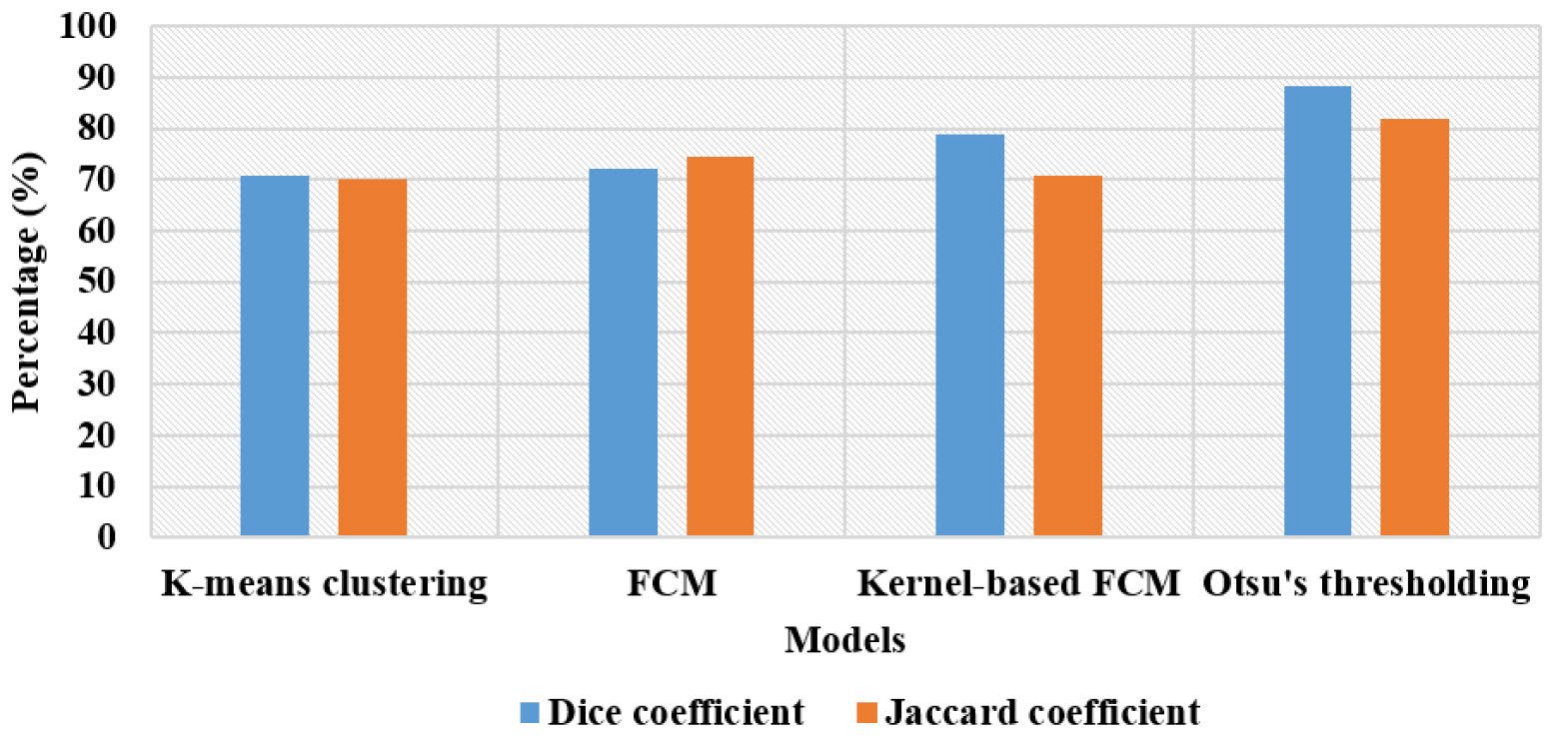
Representation of the segmentation results.

The segmentation outcomes of the proposed framework are specified in Table 1. The adopted segmentation model;Otsu’s thresholding effectiveness was compared with three existing models: FCM, K-means, and kernel-based FCM. According to Table 1, Otsu’s thresholding model obtained 88.42% of the Dice coefficient and 82.03% of the Jaccard coefficient, where the obtained results were the maximum with respect to the existing models. Otsu’s thresholding considers the maximum inter-class variance between the target images and the background region based on the threshold selection rule. The graphical representation of the segmentation results is shown in Figure 6.

**Table 1:** Segmentation results of the proposed model.

Segmentation models	Dice coefficient (%)	Jaccard coefficient (%)
K-means clustering	70.84	70.02
FCM	72.03	74.44
Kernel-based FCM	78.92	70.82
**Otsu’s thresholding**	**88.42**	**82.03**

The bold values represent the proposed model results.

The classification results of the proposed model are specified in Tables 2, 3. Particularly, the classification results of different classifiers with and without feature optimization techniques are given in Table 2. As specified there, the experimental results of different classifiers: Naïve Bayes, Random Forest, SVM, RNN, LSTM, and Bi-LSTM are given with and without utilizing a feature optimization technique called the modified genetic algorithm. After feature extraction, the direct feeding of vectors to the Bi-LSTM model obtained 98.76% of the F1 score, 97.72% of the MCC, 98.21% of sensitivity, 97.69% of classification accuracy, and 97.77% of specificity, which are all higher compared to other classifiers.

**Table 2:** Classification results by varying the classifiers.

Classifiers	F1 Score (%)	MCC (%)	Sensitivity (%)	Accuracy (%)	Specificity (%)
**Without feature optimization**					
Naïve Bayes	90.32	89.98	90.90	92.20	90.92
Random Forest	92.84	92.03	93.50	94.92	92.06
SVM	94.02	94.34	94.44	95.50	94.38
RNN	95.59	95.58	96.61	96.38	95.40
LSTM	97.60	96.07	97.90	96.95	96.55
Bi-LSTM	98.76	97.72	98.21	97.69	97.77
**With modified genetic algorithm**					
Naïve Bayes	92.30	93.29	94.30	95.40	96.84
Random Forest	94.98	96.97	96.34	96.58	96.90
SVM	97.84	97.93	97.82	97.06	97.86
RNN	98.90	98.75	98.87	98.64	98.64
LSTM	99.12	99.04	99.14	99.33	98.96
**Bi-LSTM**	**99.30**	**99.12**	**99.40**	**99.80**	**99.48**

The bold values represent the proposed model results.

**Table 3:** Classification results by varying the optimizers.

Bi-LSTM network					
Optimizers	F1 Score (%)	MCC (%)	Sensitivity (%)	Accuracy (%)	Specificity (%)
ABC	93.20	90.34	93.80	94.15	93.26
PSO	94.16	93.60	94.98	95.06	94.20
Firefly	95.26	94.74	95.70	95.98	95.43
GOA	96.55	95.44	96.44	96.95	96.58
Genetic algorithm	97.98	97.80	96.66	98.64	98.66
**Modified genetic algorithm**	**99.30**	**99.12**	**99.40**	**99.80**	**99.48**

The bold values represent the proposed model results.

**Figure 7:**
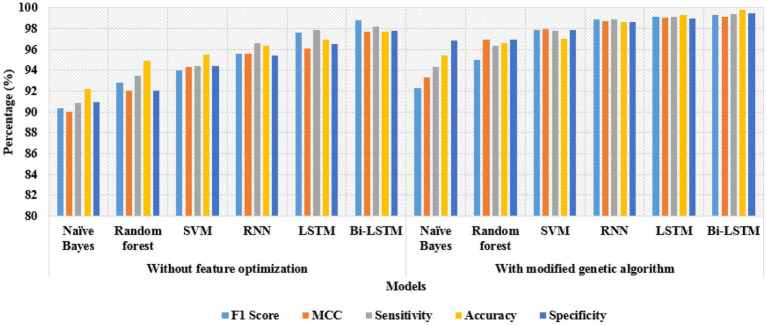
Classification results by varying the classifiers.

In addition, feeding optimal vectors selected by the modified genetic algorithm to the Bi-LSTM model achieved 99.30% of the F1 score, 99.12% of the MCC, 99.40% of sensitivity, 99.80% of accuracy, and 99.48% of specificity. The obtained experimental results are higher compared to the existing machine learning classifiers: Naïve Bayes, Random Forest, SVM, RNN, and LSTM network in intracranial hemorrhage detection. The graphical representation of the classification results by varying the classifiers is shown in Figure 7. In Bi-LSTM, the input flowed in two directions, which preserved the past and future feature information, helping to achieve better classification results.

**Figure 8:**
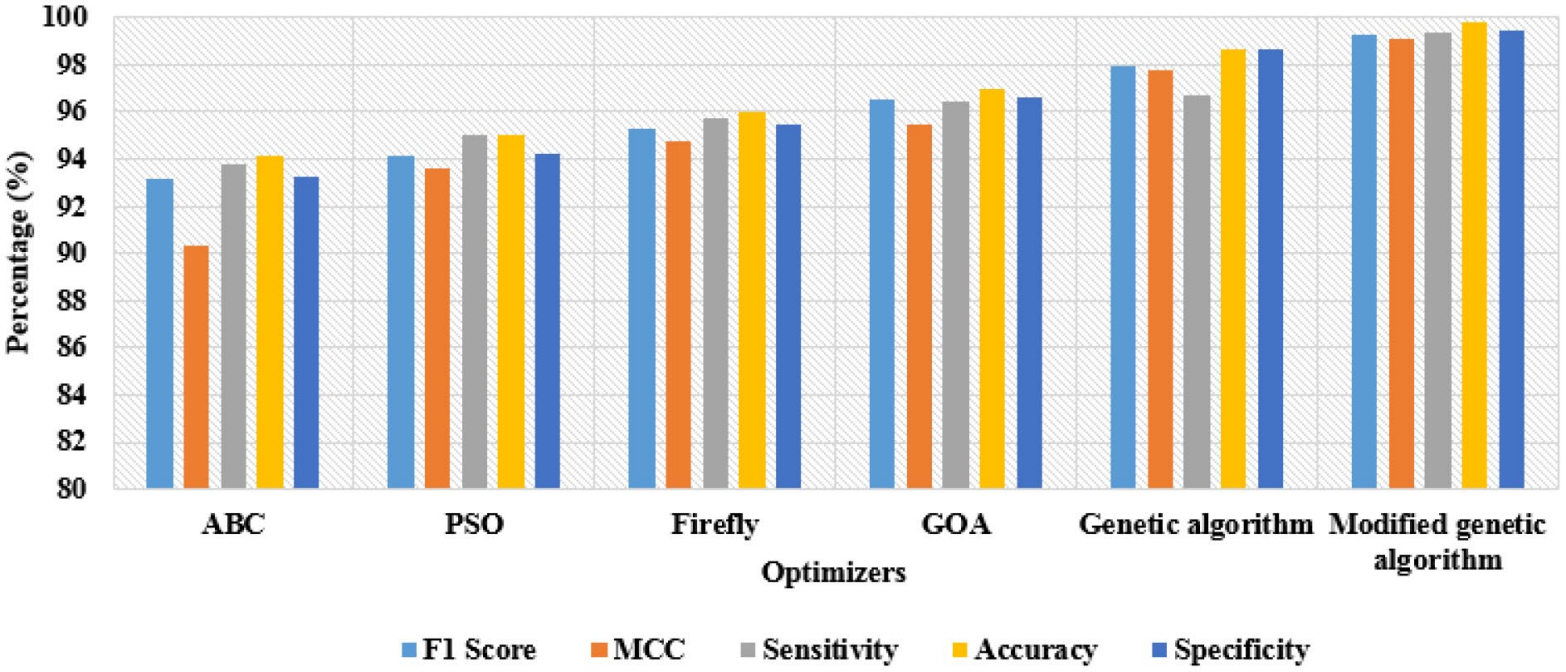
Classification results by varying the optimizers.

Additionally, the classification results obtained by varying the optimizers are represented in Table 3. In this scenario, the Bi-LSTM network is evaluated with different optimizers such as Particle Swarm Optimizer (PSO), Artificial Bee Colony (ABC), Firefly Optimizer, Genetic Algorithm, Grasshopper Optimization Algorithm (GOA), and Modified Genetic Algorithm. By looking at Table 3, the Bi-LSTM network with a modified genetic algorithm has obtained higher outcomes: 99.30% of the F1 Score, 99.12% of the MCC, 99.40% of sensitivity, 99.80% of accuracy, and 99.48% of specificity. In this research, the modified genetic algorithm effectively selected optimal vectors from the total extracted vectors, which reduced the computational complexity to linear based on the input size and order of magnitude. On the other hand, the computational time was 54.28 s, which is lower compared to the existing models. A graphical representation of the classification results by varying the optimizers is shown in Figure 8.

**Figure 9:**
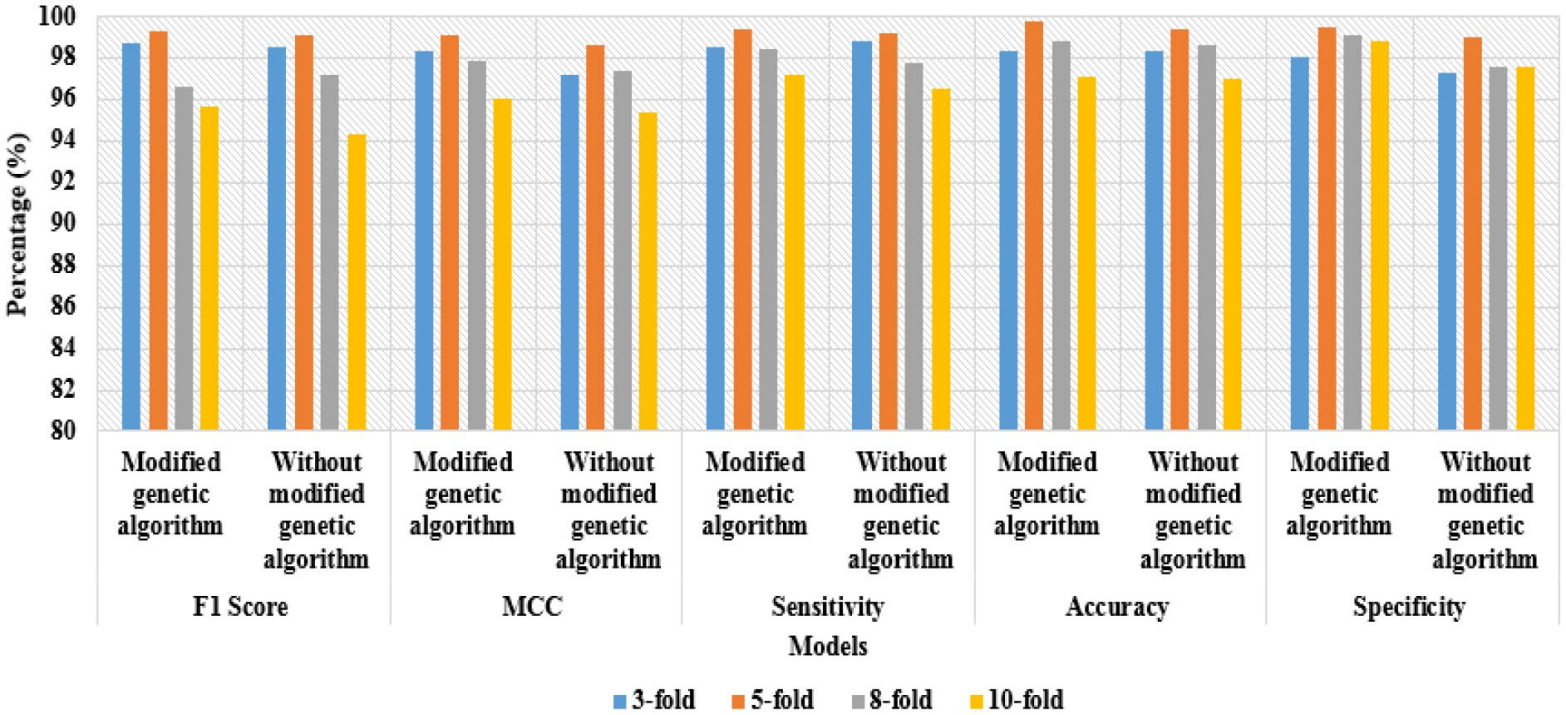
Graphical representation of the Bi-LSTM-based modified genetic algorithm for different cross-fold validations.

The experimental outcomes of the Bi-LSTM-based modified genetic algorithm for different cross-fold validations are indicated in Table 4. As shown there, the developed Bi-LSTM-based modified genetic algorithm was tested by different k-fold cross-validations, such as 3-fold, 5-fold, 8-fold, and 10-fold. According to Table 4, the Bi-LSTM-based modified genetic algorithm achieved better classification results 5-fold with respect to other cross-fold validations. In this research, the cross-fold validations improved the computational time and decreased the variance and bias of the Bi-LSTM-based modified genetic algorithm. The graphical representation of the Bi-LSTM-based modified genetic algorithm for different cross-fold validations is shown in Figure 9.

**Table 4:** Experimental outcomes of the Bi-LSTM-based modified genetic algorithm for different cross-fold validations.

K-fold cross-validations					
Evaluation metrics		3-fold	5-fold	8-fold	10-fold
F1 Score (%)	Modified genetic algorithm	98.76	**99.30**	96.60	95.68
	Without modified genetic algorithm	98.58	99.10	97.21	94.32
MCC (%)	Modified genetic algorithm	98.36	**99.12**	97.84	96.05
	Without modified genetic algorithm	97.22	98.64	97.43	95.40
Sensitivity (%)	Modified genetic algorithm	98.50	**99.40**	98.44	97.20
	Without modified genetic algorithm	98.82	99.21	97.76	96.56
Accuracy (%)	Modified genetic algorithm	98.36	**99.80**	98.86	97.12
	Without modified genetic algorithm	98.33	99.35	98.65	97
Specificity (%)	Modified genetic algorithm	98.03	**99.48**	99.14	98.80
	Without modified genetic algorithm	97.30	99	97.60	97.54

The bold values represent the proposed model results.

### 4.2. Comparative investigation

The numerical investigation between existing algorithms and the proposed Bi-LSTM based modified genetic algorithm is indicated in Table 5. Burduja et al. (2020) integrated both ResNeXt-101 and Bi-LSTM for intracranial hemorrhage detection in 3D-CT brain images. In the resulting phase, the developed deep learning model obtained 72.86% sensitivity, 97.83% accuracy, and 99% specificity. Wang et al. (2021) implemented a 2D CNN model for better detection of intracranial hemorrhage diseases. The extensive experiments confirmed that the implemented model had 95.84% sensitivity, 95% accuracy, and 94.85% specificity. Anupama et al. (2022) introduced a synergistic deep-learning model that achieved 95.73% accuracy, 97.78% specificity, and 94.01% sensitivity. Sengupta and Alzbutas (2022) combined the Optimized Gated Recurrent Unit (OGRU) with the Cuckoo Search Algorithm (CSA) for the effective detection of intracranial hemorrhage in the 3D-CT brain images. The simulation outcomes demonstrated that the ORGU-CSO model obtained 99.25% sensitivity, 99.36% accuracy, and 99.40% specificity. Asif et al. (2023) initially used the ResNet101-V2 model to extract potential vectors from the slices. Then, the Inception-V4 model was used to capture the spatial information from the second path. Finally, the outputs of the ResNet101-V2 and Inception-V4 models were fed to the light gradient boosting machine for intracranial hemorrhage detection, and the presented model achieved 96.50% sensitivity and 97.70% accuracy on the RSNA 2019 database. Regarding the comparative works, the Bi-LSTM-based modified genetic algorithm achieved high classification results in intracranial hemorrhage detection with 99.40% sensitivity, 99.80% accuracy, and 99.48% specificity.

**Table 5:** Numerical investigation between the existing and the proposed Bi-LSTM-based modified genetic algorithm.

Models	Sensitivity (%)	Accuracy (%)	Specificity (%)
ResNeXt-101 with Bi-LSTM (Burduja et al., 2020)	72.86	97.83	99
2D CNN (Wang et al., 2021)	95.84	95	94.85
Synergistic deep learning model (Anupama et al., 2022)	94.01	95.73	97.78
OGRU-CSA (Sengupta and Alzbutas, 2022)	99.25	99.36	99.40
Parallel deep convolutional model with boosting mechanism (Asif et al., 2023)	96.50	97.70	–
Bi-LSTM-based modified genetic algorithm	99.40	99.80	99.48

### 4.3. Discussion

As mentioned earlier, feature optimization and classification are integral parts of this manuscript. The proposed modified genetic algorithm used a simple entropy function to find the active vectors from the total extracted vectors. The conditional entropy value was calculated for the output vectors and the regularized vectors in the modified genetic algorithm based on an infinite feature selection technique. The modified genetic algorithm found the maximum relevance between the output vectors and the regularized vectors, which reduced the redundancy of the regularized vectors and the computation time. The computation time of the proposed model was 54.28 s, which is limited compared to the existing models. The selected vectors were fed to the Bi-LSTM model for disease-type classification. The effectiveness of the proposed framework is depicted in Tables 1–5.

## 5. Conclusion

In this study, the Bi-LSTM-based modified genetic algorithm was implemented for the early diagnosis of intracranial hemorrhage. After the acquisition of brain samples, the intracranial hemorrhage segmentation was carried out using Otsu’s thresholding technique, and further, the feature extraction was performed by combining the GLTP texture descriptor and Tamura features. The semantic gap between the extracted feature subset was reduced by integrating the global and local vectors, which improved the classification performance. Additionally, the higher-dimensional extracted vectors were reduced by proposing a modified genetic algorithm. Hence, the selection of optimal vectors or dimensionality-reduced vectors was fed to the Bi-LSTM network to classify intracranial hemorrhage sub-types (subdural, intraparenchymal, subarachnoid, epidural, and intraventricular). The extensive experiment showed that the proposed Bi-LSTM-based modified genetic algorithm achieved 99.40% sensitivity, 99.80% accuracy, and 99.48% specificity, with the achieved simulation results being higher than the comparative models, synergistic deep learning, CNN, ResNeXt-101, and ResNeXt-101 with a Bi-LSTM network. On the other hand, the Bi-LSTM-based modified genetic algorithm displayed a low computational time of 54.28 s, and the selection of discriminative vectors reduced the system complexity to linear. As a future extension, a deep learning model with an effective metaheuristic-based optimization algorithm can be developed to recognize subarachnoid hemorrhages, which could be tested in real-time on larger databases.

## Data availability statement

Publicly available datasets were analyzed in this study. This data can be found at: https://www.kaggle.com/c/rsna-intracranial-hemorrhage-detection.

## Author contributions

JS: visualization, conceptualization, formal analysis, and resources. RA: methodology, project administration, supervision, resources, investigation, and manuscript - review and editing. PF-G and BF-G: data curation, validation, and manuscript – original draft. All authors contributed to the article and approved the submitted version.

## Conflict of interest

The authors declare that the research was conducted in the absence of any commercial or financial relationships that could be construed as a potential conflict of interest.

## Publisher’s note

All claims expressed in this article are solely those of the authors and do not necessarily represent those of their affiliated organizations, or those of the publisher, the editors and the reviewers. Any product that may be evaluated in this article, or claim that may be made by its manufacturer, is not guaranteed or endorsed by the publisher.

## Figures and Tables

**Table:** 

Parameters	Definition
$\;T_{1}$	Dark regions
$\;T_{0}$	Light regions
l	Histogram bins
p(i)	Probability of distinct group
t	Threshold value
(r,c)	Rows and columns in the acquired 3D brain images
$\;\sigma_{b}^{2}\left(t\right)\;\;\;and\;\;\;\sigma_{f}^{2}\left(t\right)$	Variance of light and dark regions in the 3D brain images
$\;\mu_{b}\left(t\right)\;\;\;and\;\;\mu_{f}\left(t\right)$	Mean of light and dark regions in the 3D brain images
$w_{b}\left(t\right)\;and\;w_{f}\left(t\right)$	Weight of the light and dark regions in the 3D brain images
$\sigma_{w}^{2}$	Lower class variance
$f_{Tamura}$	Tamura features
( $G_{i},G_{j}$ )	Horizontal and vertical approximations
$G_{i,j}$	Gradient magnitude
$S_{GLTP}$	Quantized value of the neighborhood image pixels
$\;G_{c}$	Center gradient
(M,N)	Width and height of the images
γ	Classification accuracy
$T_{f}$	Extracted vectors
$l_{c}$	Chromosome length
$i_{t}$	Input gate
$f_{t}$	Forget gate
$o_{t}$	Output gate
$\;c_{t}$	Cell state
FN, TN, FP, and TP	False negative, true negative, false positive, and true positive
⊙	Pointwise multiplication
b	Bias value
$a_{t}=A\left[t,.\right]\in {\mathbb{R}}^{F}$	Quasi-periodic vectors
$h_{t-1}$	Output of the previous LSTM unit
tanh(.)	Hyperbolic tangent function
σ(.)	Sigmoid function
